# A Paradox of Genetic Variance in Epigamic Traits: Beyond “Good Genes” View of Sexual Selection

**DOI:** 10.1007/s11692-015-9359-y

**Published:** 2015-11-14

**Authors:** Jacek Radwan, Leif Engqvist, Klaus Reinhold

**Affiliations:** Evolutionary Biology Group, Faculty of Biology, Adam Mickiewicz University, Poznan, Poland; Evolutionary Biology, Bielefeld University, Bielefeld, Germany; Department of Behavioural Ecology, Institute of Ecology and Evolution, University of Bern, Bern, Switzerland

**Keywords:** Sexual selection, Mate choice, Heritability, Maintenance of genetic variance, Trade-offs, Sexual conflict

## Abstract

Maintenance of genetic variance in secondary sexual traits, including bizarre ornaments and elaborated courtship displays, is a central problem of sexual selection theory. Despite theoretical arguments predicting that strong sexual selection leads to a depletion of additive genetic variance, traits associated with mating success show relatively high heritability. Here we argue that because of trade-offs associated with the production of costly epigamic traits, sexual selection is likely to lead to an increase, rather than a depletion, of genetic variance in those traits. Such trade-offs can also be expected to contribute to the maintenance of genetic variation in ecologically relevant traits with important implications for evolutionary processes, e.g. adaptation to novel environments or ecological speciation. However, if trade-offs are an important source of genetic variation in sexual traits, the magnitude of genetic variation may have little relevance for the possible genetic benefits of mate choice.

## Introduction

Darwin (1871) developed the theory of sexual selection to explain the evolution of traits such as exaggerated antlers or bizarre plumage, which appeared inconsistent with the idea of “survival of the fittest”. Darwin argued that survival costs associated with the possession of such traits are compensated by increased success in reproductive competition, either because these traits serve as weapons in intra-sexual competition, or because they make their bearers more sexually attractive.

Maintenance of genetic variation in sexually selected traits has attracted much attention from evolutionary biologists, since prominent hypotheses for the evolution of sexual preferences assume genetic benefits for choosy females. Such indirect benefits of mate choice can stem either from positive feedback arising because the choosy sex (typically females) will produce progeny inheriting not only sexual attractiveness but also genes for preferences (Fisher 1930), or because the degree of trait elaboration indicates genetic quality (defined as breeding value for fitness (Hunt et al. 2004b) of their bearers (Zahavi 1975). Indirect genetic benefits require variation in genes underlying differential elaboration of epigamic traits (i.e. elaborated sexually selected traits associated with attractiveness), but this variation might then be expected to be depleted by the very process of sexual selection. Researchers have therefore devoted considerable attention to resolving the “lek paradox” (Borgia 1979): why are preferences maintained despite the assumed depletion of genetic variation by the very process of sexual selection?

The assumption that sexual selection depletes genetic variation was called into question by reports of substantial genetic variance in sexually selected traits (Pomiankowski and Møller 1995), a finding confirmed by recent meta-analyses (Prokop et al. 2012; Prokuda and Roff 2014). Heritabilities of traits associated with sexual attractiveness are consistently significantly above zero and, with an average of 0.48, not significantly different for comparable non-sexually selected traits (Prokuda and Roff 2014). Does this imply that the lek paradox is more apparent than real? The answer depends on the nature of genetic variation in attractiveness traits. In theory, mechanisms such as mutation-selection balance or host-parasite coevolution (Rowe and Houle 1996; Hamilton and Zuk 1982) can continuously create genetic variance in fitness, enabling genetic quality to be reflected by condition-dependent ornaments. However, Walsh and Blows (2009) have recently argued that empirically determined levels of genetic variation reported for most fitness-related traits investigated appear incompatible with strong selection reportedly acting on them. Instead, they proposed that this variance might be due to trade-offs underlying multivariate genetic constrains. Thus, in the presence of even moderate negative genetic correlations between some traits under multivariate selection, there may be little additive genetic variance (V_A_) in trait combinations associated with the direction of selection, even though there is ample V_A_ in individual traits (Walsh and Blows 2009). In this essay, we argue that trade-offs, inescapably occurring both between sexually and naturally-selected optima, and between male and female fitness, are highly relevant to understanding the maintenance of genetic variance in sexually selected traits.

**Fig. 1:**
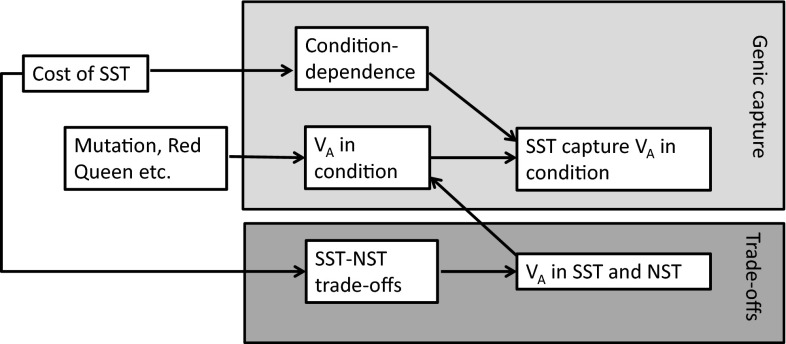
The relation between genic capture mechanism (Rowe and Houle 1996) and trade-offs hypothesis of the maintenance of genetic variance in sexual traits. Costs associated with expression of sexually selected traits (SST) have two consequences: condition-dependence of SST, and trade-offs between naturally selected traits (NST) and SST. In genic capture, SST take over genetic variance in condition, which is created independently (e.g. by influx of deleterious mutations or host-parasite arms race). We argue (lower path) that trade-offs associated with sexual traits increase the scope for the maintenance of genetic variation in sexual traits themselves, but also in other, naturally selected, traits (NST) involved in those trade-offs (see text). The observed V_A_ in sexual traits might be a combination of both mechanisms, but note that trade-offs can also contribute to V_A_ in traits affecting condition

Genetic variation in traits under sexual selection has been hypothesised to be maintained by several mechanisms (see Radwan 2008 for a comprehensive review). In order to understand how the proposed mechanisms differ in respect to the processes generating and maintaining genetic variance as well as their respective evolutionary consequences, for example regarding the benefits of mate choice, we will begin by shortly summarizing some of the most influential hypotheses. In the following sections, we will then turn our attention to the important trade-offs associated with sexually selected traits and briefly review theoretical and empirical work to demonstrate that these trade-offs are likely to increase the scope for the maintenance of genetic variation in sexually selected traits under a wide range of plausible scenarios. This implies that the sexual selection process itself can generate genetic variance in sexually selected traits (Fig. 1), and we discuss possible consequences of the nature of this variance for the evolution of sexual preferences. Furthermore, we draw attention to the underappreciated fact that trade-offs associated with the elaboration of sexually selected traits can broaden the scope for the maintenance of genetic variation also in many other, often ecologically important, traits. This is because the evolution of sexually selected traits is bound to require correlated responses in many physiological and anatomical traits (Husak and Lailvaux 2014), but these traits are also subject to multivariate genetic constraints. Standing genetic variation in these traits, increased as a by-product of sexual selection, can be a source of adaptive response to new selection pressures, with important consequences for crucial evolutionary processes, such as adaptation to new environments or speciation.

## Potential Mechanisms Maintaining Genetic Variation Under Sexual Selection

### Condition-Dependence and Genic Capture

While the complexity of the genetic underpinnings of sexually selected traits (e.g. a simple morphological trait such as an elongated tail) is presumably usually lower than that of life-history traits, they are nevertheless costly to produce. Thus the elaboration of a sexual trait should depend on the resources available to an individual. Based on this, the “genic capture” hypothesis posits that genetic variation in the expression of sexual traits represents genetic variation in resource acquisition and utilisation efficiency, or “condition” (Andersson 1986; Rowe and Houle 1996). This variation may be substantial because condition is likely to be affected by many traits, and thus many genes, making it a large target for deleterious mutations. This leads to the prediction that, via condition-dependence, sexual trait should show high sensitivity to mutations, evidence for which so far is scarce (Pekkala et al. 2009). V_A_ in condition can also be due to mechanisms other than mutations, e.g. by genes responsible for parasite resistance, potentially leading to a polymorphism that is maintained by host-parasite co-evolutionary cycles (Hamilton and Zuk 1982; Milinski 2006).

### Modifier Selection

Another hypothesis was put forward by Pomiankowski and Møller (1995), who proposed that if selection on sexually selected traits is greater than linear (i.e. when the second derivative of the fitness function is positive), increased phenotypic variance in these traits will be selected for. Consequently, modifier genes increasing genetic variance in epigamic traits will be favoured. This model was criticized because the validity of the assumption of an accelerating fitness function for sexually selected traits has not been demonstrated, selection on modifiers is weak and, apart from a period during their initial evolution, sexually selected traits are likely to be under net stabilizing, rather than directional selection (Rowe and Houle 1996). A related hypothesis by Petrie and Roberts (2007) proposed that mutator genes may increase in frequency under sexual selection, but this requires linkage between the mutator and a gene affecting the sexually selected trait.

### Balancing Selection

Balancing selection may result from several mechanisms, such as heterozygote advantage, negative-frequency-dependent selection or antagonistic pleiotropy (reviewed in Radwan 2008). Balancing selection may operate directly on sexually selected traits, e.g. frequency-dependent selection appears to favour rare colour patterns in male guppies (Hughes et al. 2013), but there are no particular reasons to expect similar mechanisms to be widespread. Frequency-dependence is more likely to act on traits affecting condition, especially in the form of parasite-driven selection on rare immune genes (Milinski 2006; but see Hughes 2015). However, antagonistic pleiotropy is potentially the most widespread mechanism of balanced polymorphism associated with sexual selection because alleles affecting reproductive success by varying the expression of sexual ornaments will simultaneously affect other life-history traits, e.g. survival due to increased visibility to predators. Another, equally pervasive trade-off is associated with sexual antagonism (or intersexual ontogenetic conflict, Rice and Chippindale 2001): genes associated with male sexually selected traits may have negative effects when expressed in females (see below for more details).

Conditions for antagonistic pleiotropy to maintain stable polymorphism have been considered restrictive, because they require beneficial reversal of dominance (Curtsinger et al. 1994). This scepticism is somewhat surprising given that the famous case of heterozygote advantage, sickle-cell anaemia, can also be interpreted as an example of antagonistic pleiotropy between two fitness-related traits: parasite resistance and physiological efficiency (Hill et al. 1991). In a similar vein, Fry (2010) pointed out that dominance of an allele with respect to a particular trait is not necessarily the same as dominance with respect to fitness accrued via this trait (or sex), and argued that if mapping of fitness to a trait is concave, beneficial reversal with respect to fitness is easily achieved. Furthermore, Sellis et al. (2011) and Connallon and Clark (2014a) emphasized that most of the above mentioned theory cannot predict the probability of balancing selection because it does not incorporate details of the fitness effect of alleles and genotypes, which determine parameter space available to selection. Building on Fisher’s geometric model of multivariate adaptation to assess these effects, Connallon and Clark (2014a) concluded that sexual antagonism causes these fitness effects to occupy the portion of parameter space most favourable for balancing selection.

Overall, some mechanisms, e.g. modifier selection, can maintain V_A_ specifically in sexually selected traits. Other mechanisms, such as mutation-selection balance or host-parasite cycles, can maintain variation in total fitness, which can be reflected by condition-dependent sexual ornaments. However, mechanisms such as negative pleiotropy or sexual antagonism will maintain V_A_ in sexually selected traits and other fitness-related traits, but little V_A_ in overall fitness (Pischedda and Chippindale 2006; Delcourt et al. 2012). Under these mechanisms, there are no ‘good’ and ‘bad’ genes, but alternative routes to equal fitness.

## The Role of Trade-Offs in Explaining the Paradox of High Genetic Variance in Sexually Selected Traits

There is now increasing appreciation of the role that fitness trade-offs (manifested at the genetic level as antagonistic pleiotropy) play in maintaining genetic variation either via balancing selection, or by affecting mutation-selection balance (Connallon and Clark 2012). As mentioned above, two types of trade-offs are especially likely to arise because of the action of sexual selection: one between sexually and naturally selected traits within the sex expressing the trait, and another associated with the expression of the same genes in males and in females. The former, associated with the production of costly sexually selected traits, is likely to be widespread (e.g. Brooks 2000; Hunt et al. 2004a)—indeed it motivated Darwin to develop the theory of sexual selection. The latter is termed intra-locus sexual conflict (reviewed in Bonduriansky and Chenoweth 2009), also referred to as sexual antagonism (Connallon and Clark 2014b), and arises when different alleles at a single locus have opposing effects on male and female fitness (reviewed in Bonduriansky and Chenoweth 2009). Sexual antagonism is also expected to be widespread: under Fisher’s geometric model of multivariate adaptation, it inevitably arises under a wide range of conditions (Connallon and Clark 2014a). Estimates in *Drosophila melanogaster* suggest that over 60 % of genes affecting fitness exhibit such genetic conflicts. Sexual selection is likely to play a prominent role in causing differences in male and female fitness landscapes. Indeed, Cox and Calsbeek (2009) found that sexual selection results in disparate selection gradients acting on males and females more often than fecundity or viability selection.

Recent findings show that increased sexually selected dimorphism is associated with elevated sex-bias in gene expression in a large number of genes (Pointer et al. 2013; Stuglik et al. 2014). This is an expected consequence of sexual antagonism, and also implies a major role of sexual selection as the source of the conflict. While sex-biased trait expression is thought to alleviate intra-locus sexual conflict, potentially even leading to positive correlations between sexual dimorphism and fitness (Arnqvist and Tuda 2010), several recent studies (Harano et al. 2010; Plesnar Bielak et al. 2014) demonstrated that an elaboration of sexually selected traits is nevertheless genetically negatively correlated with female fitness. These findings indicate that despite selection for conflict resolution, sexually-antagonistic genetic variation apparently still segregates within natural populations. Based on recent theory, this is expected (Connallon and Clark 2014a): opportunities for balancing selection increase due to trade-offs associated with sexual antagonism which inevitably arises from of sexually selected dimorphism.

While the potential for trade-offs between sexually and naturally-selected optima to maintain genetic variation has, to our knowledge, not been explicitly modelled, the principles leading to the maintenance of polymorphism as demonstrated for sexual antagonism (see e.g. Fry 2010; Connallon and Clark 2012; Arnqvist et al. 2014; Kidwell et al. 1977) are likely to apply to a broader class of fitness trade-offs (see Connallon and Clark 2012, 2014a for discussion). The potential of such trade-offs to maintain genetic polymorphism in sexually selected traits is illustrated by a recent study in Soay sheep, which characterised genetic variation in sexually selected horn size. This variation is due to a polymorphism at a single gene, relaxin-receptor 2 (RXFP2), explaining 76 % of additive genetic variation (V_A_) in horn size. Horned males have higher reproductive success, but lower survival (Johnston et al. 2013). The dominance in this allelic system is reversed in favour of higher heterozygote fitness: reproductive success of heterozygotes is similar to that of homozygotes carrying the allele for big horns, but in terms of survival they are close to the homozygote for reduced horns.

It is also worth stressing that even if intra-locus sexual conflict is resolved and genes or traits become sex-limited in expression, genetic polymorphism might still be an expected outcome. The crucial ingredient in this case is fluctuating selection over time (Reinhold 2000; Gorelick and Bertram 2003). While conditions for polymorphism are generally difficult to achieve under such temporal fluctuations (see e.g. Hedrick 1986), they get much broader if traits are sex-limited. The reason is that genes that are temporarily suboptimal in a certain sex will be shielded from selection in the sex where they are not expressed and this may well lead to stabilizing a genetic polymorphism over time. Fluctuating selection, evident for instance from the widespread existence of adaptive genotype × environment interactions, seems to be widespread for sexually selected traits (Qvarnström 1999; Bertram 2002; Danielson-Francois et al. 2006; Ingleby et al. 2010).

Strong sexual selection acting on males can enhance the scope for the maintenance of polymorphism even further due to narrowing the distribution of optimal values of the male-benefit phenotype. To see why, consider that sexual antagonism can arise due to gene-by-sex interactions (a variant of gene-by-environment interaction), resulting from sexually dimorphic effects of alleles on female and male phenotypes (Connallon and Clark 2014b). This is analogous to a scenario where individuals experience different habitats or patches that vary in their trait optima (see Frank and Crespi 2011; Haig et al. 2014). In these situations, a polymorphism is easier to achieve when the relative distance in optimal traits values between the two habitats—or in this case, between the two sexes—is large (Hedrick 1986; Kisdi and Geritz 1999; Felsenstein 1976; Levene 1953). Strong sexual selection should make the distribution of antagonistic trait values narrower in males because typically only a small fraction of males will reproduce—in particular those with traits closest to the male optimum. Consequently, male and female distributions overlap less and it will be easier to achieve the condition necessary to maintain a polymorphism.

Finally, apart from broadening the scope for balanced polymorphisms, trade-offs associated with the evolution of sexually selected traits result (on average) in weaker selection against deleterious mutations. Consequently, sexually-antagonistic mutations are expected to segregate in populations for longer than mutations deleterious for both sexes, resulting in larger amounts of genetic variance segregating in populations (Connallon and Clark 2012).

In summary, it appears that trade-offs between male sexually-selected traits and ecologically selected traits (including female traits) unlock a number of ways by which genetic variance can be maintained. In the next two sections we consider the consequences of the nature of that variation for sexual and natural selection.

## Consequences for Understanding the Process of Sexual Selection

One of the leading hypotheses proposed to resolve the lek paradox is the genic capture mechanism (Rowe and Houle 1996), which posits that given condition-dependence of sexually selected traits, considerable genetic variance in them can be maintained because condition depends on many genes affecting resource acquisition and processing. However, genetic benefits of mate choice can arise only as long as genetic variance in condition reflects variance in fitness, as would be the case, for example, when it is caused by continuous influx of unconditionally deleterious mutations, or by host-parasite coevolutionary cycles (Hamilton and Zuk 1982).

On the other hand, if the substantial genetic variance observed in sexually selected traits results mostly from trade-offs inevitably associated with their evolution, more elaborated traits will not be associated with higher fitness (i.e. most genetic variation will be orthogonal to the direction of selection (Walsh and Blows 2009; Delcourt et al. 2012), implying that there will be little genetic benefits of mate choice. For example in red deer (Kruuk et al. 2002) and guppies (Hall et al. 2004), no response to selection on heritable sexual ornaments was observed. One explanation for this paradoxical result is negative genetic correlation between male sexually selected traits and female fitness (Hall et al. 2004; Foerster et al. 2007).

Furthermore, even if genetic benefits of mate choice are non-negligible, trade-offs associated with the evolution of sexually selected traits can affect the evolution of female preferences in interesting ways. This is exemplified by recent modelling, which assumed a trade-off between the elaboration of traits used in sexual competition and the ability to utilize resources (a simple example of such a trade-off would be enhanced reproductive success but reduced survival associated with elaborated ornamentation). If benefits to females of mating with the most competitive males were assumed to be large, extreme values of male traits evolve. In case of small benefits, arguably a more likely situation in case of genetic benefits of mate choice, trade-offs caused cyclical dynamics of sexually selected traits, such that their elaboration can decrease at some points of the cycle, followed by directional selection leading again to extreme trait elaboration. Under this scenario, preferences for epigamic traits easily evolve and are maintained in populations (Baldauf et al. 2014). Intriguingly, these dynamics can even lead to substantial polymorphism in female preferences, such that even if on average elaborate epigamic traits are preferred, some females prefer males with the least-elaborated traits (Baldauf et al. 2014). This implies that selection acting on female preferences may be more complex than so far assumed.

## Sexual Selection Can Generate Variation in Ecologically-Relevant Traits

If, as argued above, trade-offs between sexually selected and naturally-selected optima help maintain genetic variation, this variation will necessarily be maintained in both sexually selected and ecologically selected traits. Furthermore, elaboration of costly sexually selected traits causes correlated selection on other traits (Husak and Lailvaux 2014), the expression of which can be subject to sexual antagonism or life-history trade-offs. These traits will often be highly ecologically relevant, e.g. when sexual selection generates sexually-antagonistic selection on genes affecting metabolic processes (Stuglik et al. 2014). A recent meta-analysis (Prokop et al. 2012) found that sexual attractiveness is associated with higher values of various ‘performance traits’ such as metabolic efficiency or immune response, indicating that sexual selection indeed acts on these ecologically important traits. Hence, these trait can be subject to similar trade-offs (including sexual antagonism) as classical sexually selected traits, and genetic variance in them should be maintained by the same processes which were discussed above.

While under constant selection this variance is likely to be orthogonal to the direction of selection (Walsh and Blows 2009; Delcourt et al. 2012), it might be ‘released’ and facilitate the response to selection when environmental change alters the shape of the fitness landscape. For example, a recent study by Hollis et al. (Hollis et al. 2014) showed that substantial sexually-antagonistic genetic variation does indeed segregate in populations, as evidenced by the rapid evolution of gene expression patterns from a male to a female optimum when sexual selection acting on males was minimized.

## Testing Predictions

The basic prediction we propose to test is that stronger sexual selection is associated with increased genetic variation. One way of achieving this would be to use comparative approaches. The direct tests are yet to be performed, but some indirect support for this prediction comes from association between sexual dichromatism (used as a proxy for the strength of sexual selection) and species turnover rate (Doherty et al. 2003). Higher extinction rate can be explained e.g. by increased predation risk associated with conspicuous colouration or sexual antagonism negatively affecting population productivity. However, the improved ability of dichromatic species to colonize new patches of habitat, which fully compensated the increased extinction rate, can be explained by higher amount of standing genetic variation segregating in species under stronger sexual selection, which facilitates adaptation of these species to novel environments. The same explanation can be applied to a finding that bird species under stronger sexual selection evolved earlier arrival on breeding sites in response to global warming compared to species with less intense sexual selection (Spottiswoode et al. 2006). Nevertheless, this pattern can also be explained by a ‘good genes’ scenario (Candolin and Heuschele 2008). Comparing the amount of genetic variance between more and less dimorphic populations or species would help to discriminate between these alternatives.

Further tests could be provided by experimental evolution. While several studies have manipulated the opportunity of sexual selection to explore whether it facilitates adaptation to novel environments, their design was based on the assumption that sexual selection simply strengthens natural selection, and thus the rate of adaptation to a novel environment (Candolin and Heuschele 2008; Plesnar-Bielak et al. 2012). Consequently, environment and mating systems are typically manipulated simultaneously. In contrast, we predict that stronger sexual selection will result in increased genetic variance in sexually selected and ecological traits. To test this, V_A_ in these traits should be measured, and adaptation to novel environments should be investigated after prolonged periods of evolution under varying intensity of sexual selection.

## Other Evolutionary Consequences and Outstanding Questions

Increased genetic variance in sexually selected traits and in the traits involved in the associated trade-offs, may have consequences for a number of important evolutionary processes. We here shortly highlight several potentially profound implications.

### Evolution of Mating Preferences

Consequences of the role of sexual selection in generating genetic variance in sexually selected and ecological traits need to be explored. The consequences of spatial/temporal variation in ecological conditions for evolution of mating preferences seem particularly interesting.

### Adaptability

Adaptation often occurs based on standing genetic variation; by maintaining genetic variation in ecologically relevant traits, sexual selection may facilitate adaptation to novel/changing environments.

### Population Extinction

Theory (Kokko and Brooks 2003) predicts that elaboration of sexually selected traits at the cost of viability may lead to extinction especially when environmental conditions deteriorate. However, if negative pleiotropy maintains considerable genetic variance, populations can quickly adapt to deteriorated conditions (at the cost of sexually selected traits).

### Speciation

Maintenance of genetic variance by sexual selection is likely to facilitate local adaptation, a pre-requisite of ecological speciation. Because of trade-offs involved, changes in ecological conditions are likely to change the optimal value of a sexually selected trait, and possibly also of female preferences. This may provide a new link between ecology and mating preferences, facilitating speciation (Weissing et al. 2011).

### Evolution of Sex

Good genes models of sexual selection have shown that sexual selection can reduce the cost of sex because it facilitates purging of deleterious mutations form populations (Agrawal 2001; Siller 2001; but see Connallon et al. 2010). In contrast, the view of sexual selection that we outline here implies that it may facilitate adaptation to a novel environment because it will increase overall genetic variability and hence evolvability.

## Concluding Remarks

We argue that trade-offs associated with sexually selected traits imply that genetic variation in these traits is likely to increase as they become more elaborate. This way, the opportunity for the maintenance of variation is created by the evolution of sexually selected traits themselves, rather than by sexually selected traits capturing the existing variation of other traits. The consequences for the evolution of mating preferences need to be further explored. On the one hand, variation arising due to multivariate genetic constraints is likely to allow for little benefits of mate choice (Hine et al. 2004; Delcourt et al. 2012). On the other hand, in combination with even small genetic benefits of mate choice, trade-offs can lead to unexpected dynamics of evolution of sexually selected traits and female preferences, with the possibility of genetic polymorphism in the latter (Baldauf et al. 2014).

Importantly, the evolution of increased sexual competitiveness (including elaboration of classical epigamic traits) is likely to impose antagonistic selection on many genes involved in basic organismal processes. Consequently, in as much as such trade-offs contribute to the maintenance of genetic variation (Connallon and Clark 2014a; Johnston et al. 2013; Fry 2010), sexual selection should result in increased genetic variance in many ecologically relevant genes, which can have broad implications for population evolvability and adaptability. This can be another, yet unconsidered (Bonduriansky 2011), pathway by which sexual selection contributes to local adaptation and ecological speciation.

Conversely, fluctuations in ecological conditions can have consequences for the evolution of sexually selected traits and mate choice that are also worth exploring more fully. In our opinion, the realisation of the consequences of sexual selection for the maintenance of genetic variance in sexually selected traits opens new research perspectives in several areas of evolutionary biology.
